# Author Correction: Immediate neural impact and incomplete compensation after semantic hub disconnection

**DOI:** 10.1038/s41467-023-43811-0

**Published:** 2023-12-04

**Authors:** Zsuzsanna Kocsis, Rick L. Jenison, Peter N. Taylor, Ryan M. Calmus, Bob McMurray, Ariane E. Rhone, McCall E. Sarrett, Carolina Deifelt Streese, Yukiko Kikuchi, Phillip E. Gander, Joel I. Berger, Christopher K. Kovach, Inyong Choi, Jeremy D. Greenlee, Hiroto Kawasaki, Thomas E. Cope, Timothy D. Griffiths, Matthew A. Howard, Christopher I. Petkov

**Affiliations:** 1 Department of Neurosurgery, University of Iowa, Iowa City, IA USA; 2 Biosciences Institute, Newcastle University Medical School, Newcastle upon Tyne, UK; 3 Neuroscience Institute, Carnegie Mellon University, Pittsburgh, PA USA; 4 Departments of Neuroscience and Psychology, University of Wisconsin, Madison, WI USA; 5 CNNP Lab, Interdisciplinary Computing and Complex BioSystems Group, School of Computing, Newcastle University, Newcastle upon Tyne, UK; 6 UCL Institute of Neurology, Queen Square, London, UK; 7 Department of Psychological and Brain Science, University of Iowa, Iowa City, IA USA; 8 Psychology Department, Gonzaga University, Spokane, WA USA; 9 Department of Radiology, University of Iowa, Iowa City, IA USA; 10 Iowa Neuroscience Institute, University of Iowa, Iowa City, IA USA; 11 Department of Communication Sciences and Disorders, University of Iowa, Iowa City, IA USA; 12 Department of Clinical Neurosciences, Cambridge University, Cambridge, UK; 13 MRC Cognition and Brain Sciences Unit, Cambridge University, Cambridge, UK

**Keywords:** Language, Cortex, Perception

Correction to: *Nature Communications* 10.1038/s41467-023-42088-7, published online 07 October 2023

In this article Thomas E. Cope, Timothy D. Griffiths, Matthew A. Howard III and Christopher I. Petkov should have been denoted as equally contributing joint senior authors. The original article has been corrected.

