# Supplementary material for: Immediate neural impact and incomplete compensation after semantic hub disconnection
Source: Nat Commun. 2023 Oct 7;14:6264. doi: 10.1038/s41467-023-42088-7 (PMC10560235; doi:10.1038/s41467-023-42088-7)
Supplement: Supplementary file 1 — Supplementary Information [file 41467_2023_42088_MOESM1_ESM.pdf]

**SUPPLEMENTARY MATERIALS**

**FIGURES**

**Suppl. Fig. 1:** Behavioral /b/ bias condition results

**Suppl. Fig. 2:** ATL connectivity with frontal and temporal areas

**Suppl. Fig. 3:** Resection impact on cortical tissue, connectivity matrix similarity across participants and P1 simulated changes to connectome only including TP impact

**Suppl. Fig. 4:** Disconnection speech response impact on HG, IFG and STG across frequency bands

**Suppl. Fig. 5:** Speech predictability mismatch effects in HG, STG and IFG

**Suppl. Fig. 6.** Mismatch response evaluated with different baselining methods.

**Suppl. Fig. 7:** Temporal Pole (TP) pre-disconnection Conditional Granger Causality (CGC) results

**Suppl. Fig. 8:** ATL disconnection impact on fronto-temporal network effective connectivity

**TABLES**

**Suppl. Table 1:** Results of logistic mixed effects model for the behavioral task for each participant

**Suppl. Table 2:** Pre- and post-operative neuropsychological testing scores and calculated Reliable Change Index (RCI) for P1 and P2

**Suppl. Table 3:** VOT peak times on the averaged and individual ERPs on the Heschl's gyrus electrodes pre- and post-disconnection

**Suppl. Table 4:** Physiological parameters during the pre- and post-disconnection recording periods

**Suppl. Table 5:** Detailed description of the experimental stimuli

**Suppl. Table 6:** Detailed results for the hubness strength of TP electrodes

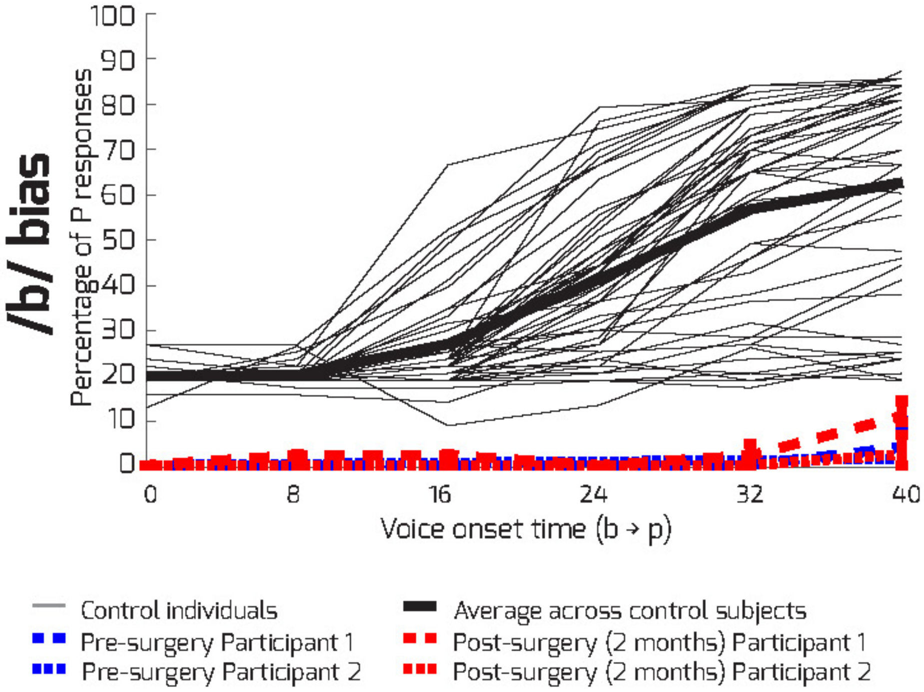


**Supplementary Figure 1. Behavioral /b/ bias condition results.** Percentage of P responses to the /b/ bias words in relation to the VOTs (from /b/ to /p/ sounds) from the control participants and P1 and P2. The patient data were obtained 2-6 weeks before and two months after their surgery. Format as in manuscript Fig. 1F. The /p/ bias responses (manuscript Fig. 1F) are well within the range of responses of the control participants' data pre-disconnection and are substantially disrupted post-disconnection in both P1 and P2. The /b/ bias responses of both participants shown here are outliers both before and after the surgery.

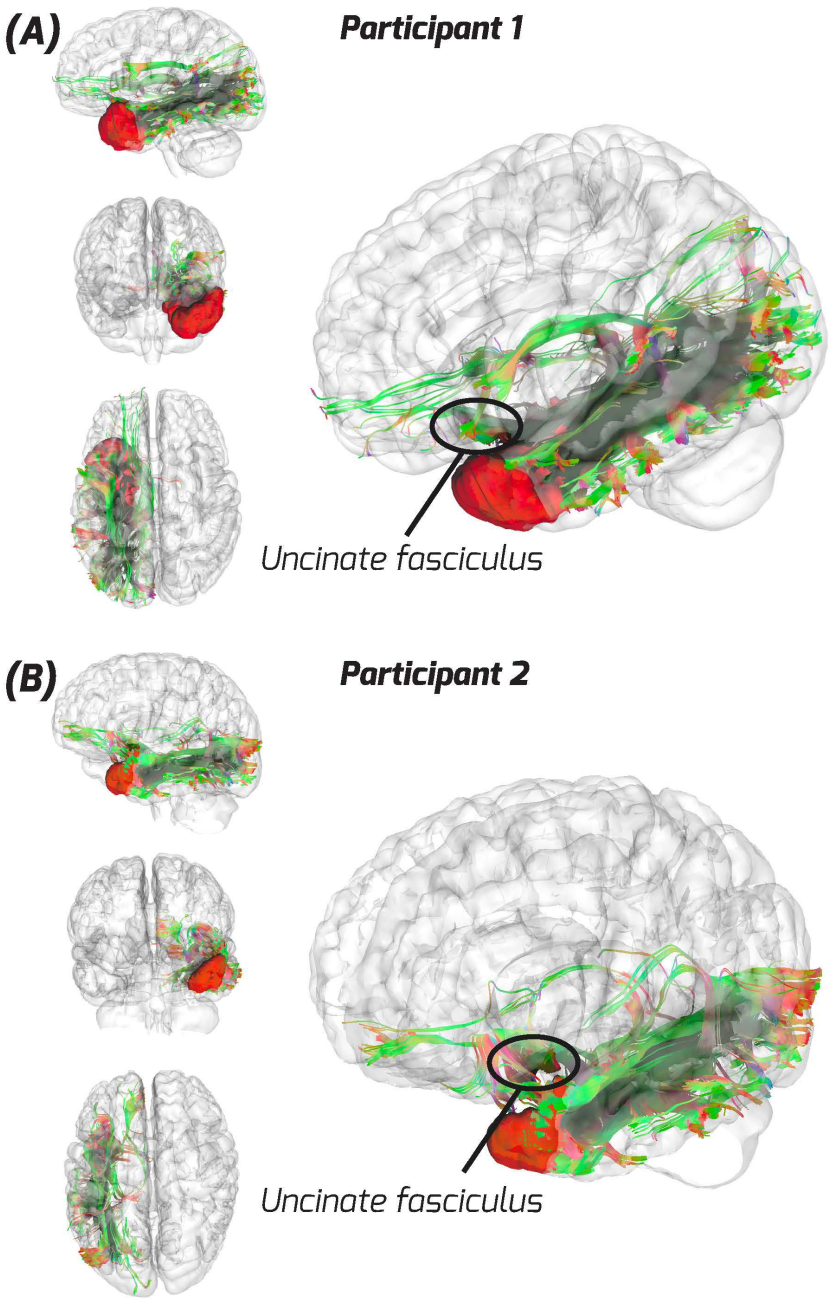


**Supplementary Figure 2. ATL connectivity with frontal and temporal areas before the surgery.** The figure shows the streamlines connecting ATL to various frontal and temporal regions based on the pre-surgical diffusion-weighted MRI (dMRI) for Participant 1 (panel A) and participant 2 (panel B). The indicated area shows the presence of the uncinate fasciculus connecting the ATL to the inferior portions of the frontal lobe, as well as white matter tracts interconnecting the ATL with more caudal parts of the temporal lobe. Images created from individual dMRI images with DSI studio[1].

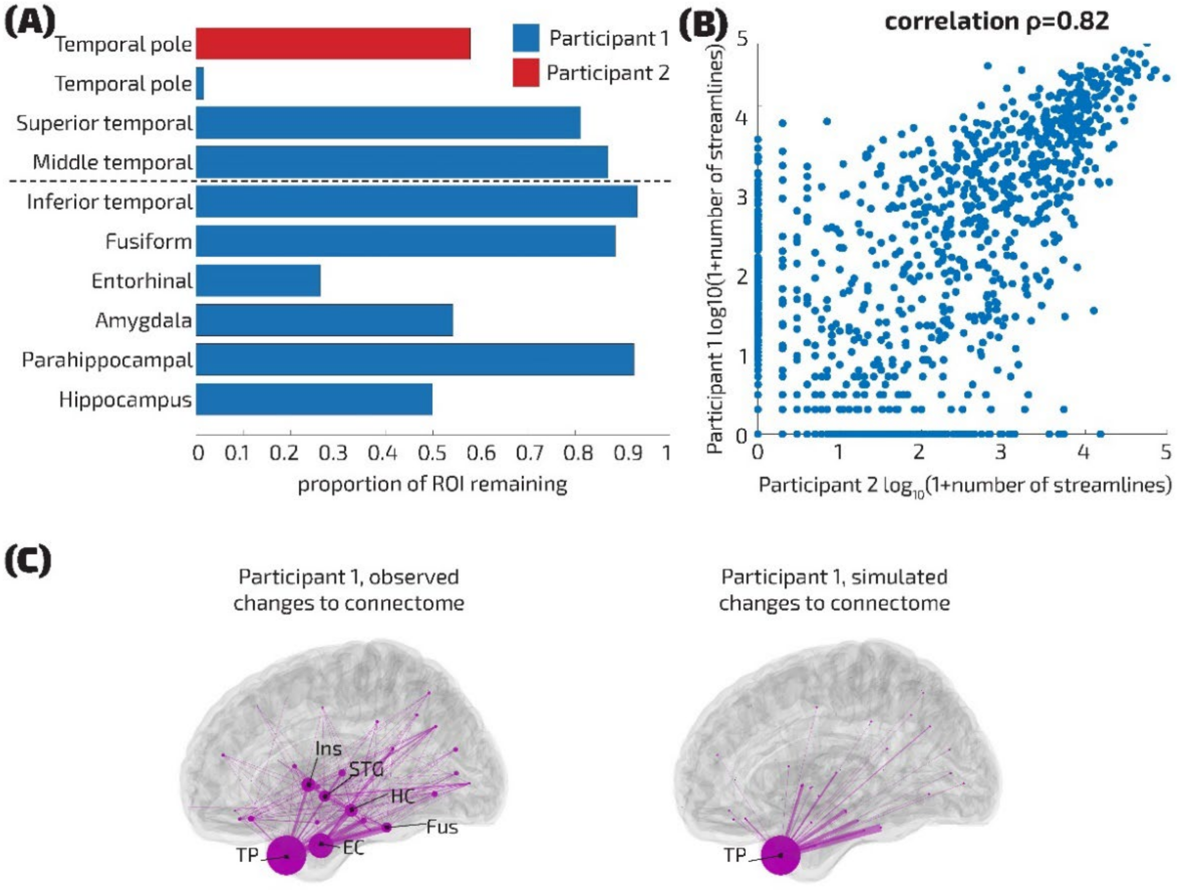


**Supplementary Figure 3. Resection impact on cortical tissue, pre- and post-surgical diffusion MRI connectivity matrix similarity across participants, and P1 simulated changes to connectome only including TP impact.** (A) The amount of the given region left after the entire surgical procedure based on the T1-weighted scans recorded before and two months after the disconnection of the left ATL for P1 and P2. P1's entire resection procedure involved both the ATL disconnection and para/hippocampal resection (blue bars). The dashed line denotes the separation of the two steps of the surgical procedure: in step 1, the ATL is disconnected, and the superior and middle temporal gyri are also affected (after this step the post ATL disconnection dataset was collected); in step 2, deeper structures are resected with the following brain regions affected: inferior temporal and entorhinal cortex, fusiform and parahippocampal gyrus, amygdala, and hippocampus. P2's procedure only involved the ATL disconnection affecting the TP (red bar). (B) Similarity of connectivity matrices before disconnection. Shown is the comparison of P1 and P2's log transformed number of streamlines between the nodes in their connectome, based on the dMRI images before disconnection. (C) Observed reduction (left) in region-region streamlines (proportional to line width) and hubness (degree centrality; proportional to node diameter) in P1 after ATL and para/hippocampal resection (TP: temporal pole; Ins: insula; STG: superior temporal gyrus; HC: hippocampus; EC: Entorhinal cortex; Fus: Fusiform gyrus). Also shown is the simulated structural impact of disconnecting only the TP node (right). The empirically observed impact includes the expected reduction in TP centrality seen in the simulation, and all edges disconnected within the

simulation were also disconnected, featuring prominently in the observed impact. The simulated partial surgical impact is therefore consistent with the observed post-surgical impact, and plausibly representative of the status of the disconnection at the time of the functional recordings.

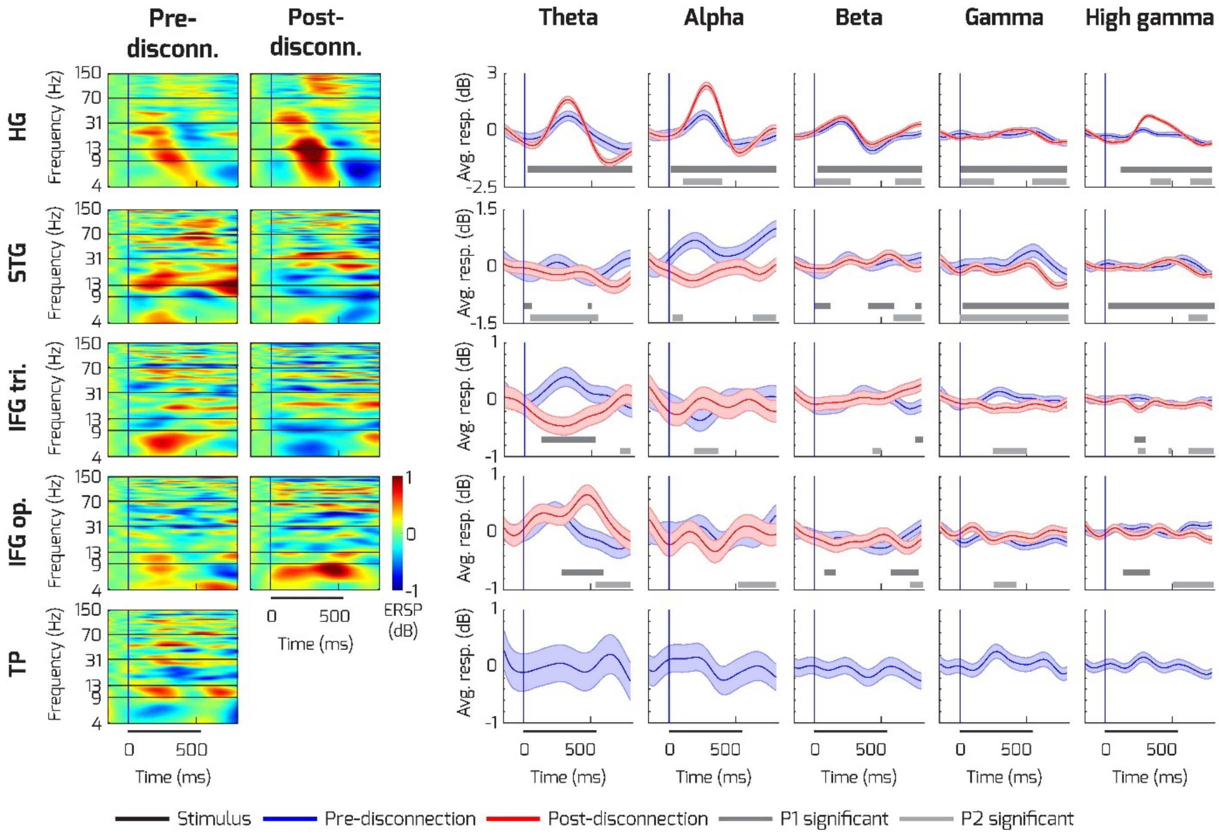


**Supplementary Figure 4. Disconnection speech response impact on HG, IFG, STG and TP across frequency bands.** Format as in manuscript Fig. 2B. Left two columns: Event-related spectral perturbation responses to the target speech sound (stimulus onset indicated by black bar below plots) in HG, STG, IFG pars triangularis and opercularis and TP electrodes for both participants. TP contacts were only neurophysiologically viable pre-disconnection. Right: Averaged theta (4-8 Hz), alpha (9-12 Hz), beta (13-30 Hz), gamma (31-69 Hz) and high gamma (70-150 Hz) frequency band responses plotted together with the standard error of mean (SEM). Blue lines show the responses before, red lines show the responses after disconnection of the ATL. Gray bars indicate permutation tested significant differences post- vs pre-disconnection for P1 and P2, respectively (cluster-based permutation test $p < 0.05$ for at least 25 ms time windows). Unlike HG which shows substantial magnification of speech responses (also see manuscript Fig. 2B), STG shows primarily disruption of speech representations post-disconnection. IFG pars opercularis shows theta speech response enhancement after disconnection.

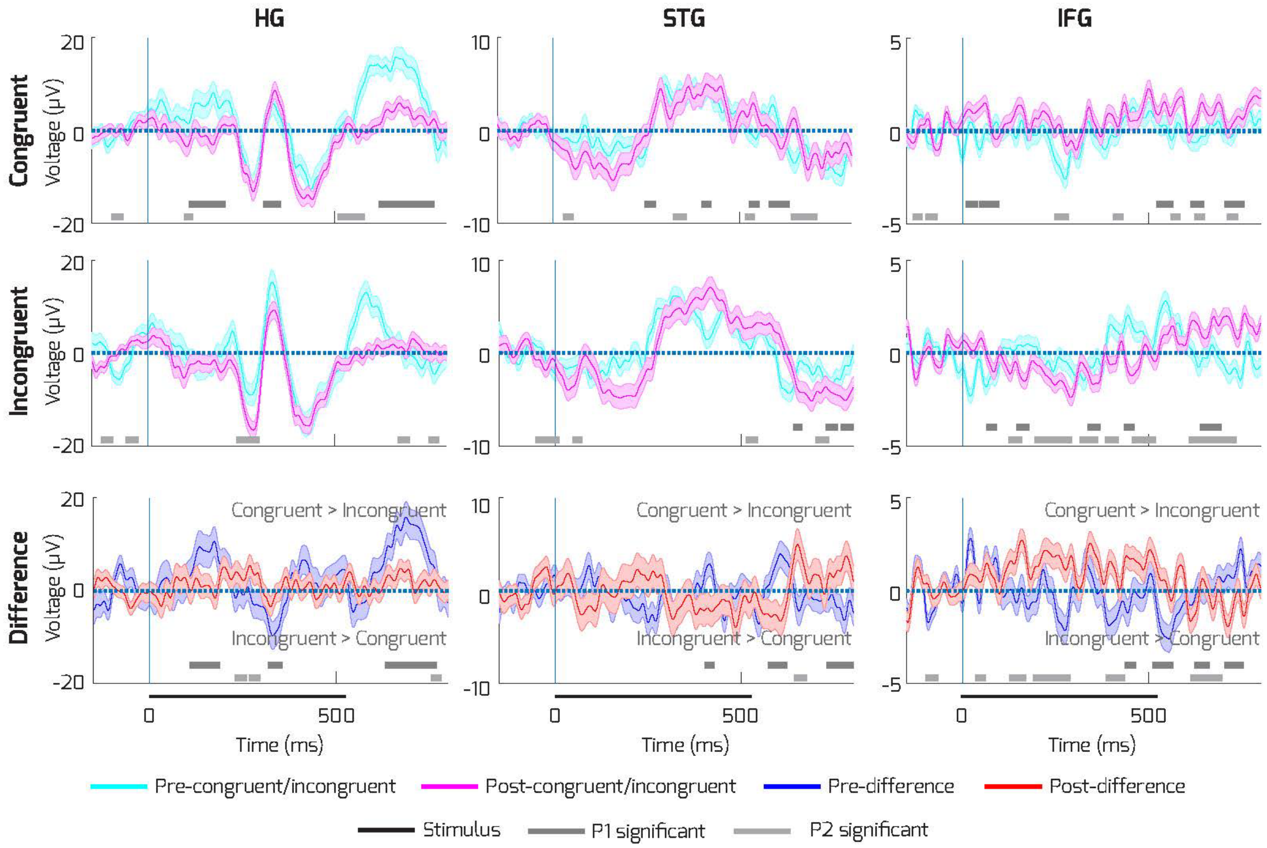


**Supplementary Figure 5. Speech predictability mismatch effects in HG, STG and IFG.** Top row shows the responses to the congruent condition before (cyan) and after (magenta) the disconnection. The second row shows the same but for the incongruent condition. The third row shows the contrast between congruent and incongruent conditions before (blue) and after (red) the disconnection. The solid black line under the time axis depicts the average length of the target speech stimuli. Gray bars indicate permutation tested significant differences post- vs pre-disconnection for P1 and P2, respectively (cluster-based permutation test $p < 0.05$ for at least 25 ms). Format as in manuscript Fig. 2C. HG congruency effects are strong in HG and disrupted post-disconnection (bottom left). Such effects are weaker in STG and IFG but show some consistent disruption in both participants at later timepoints.

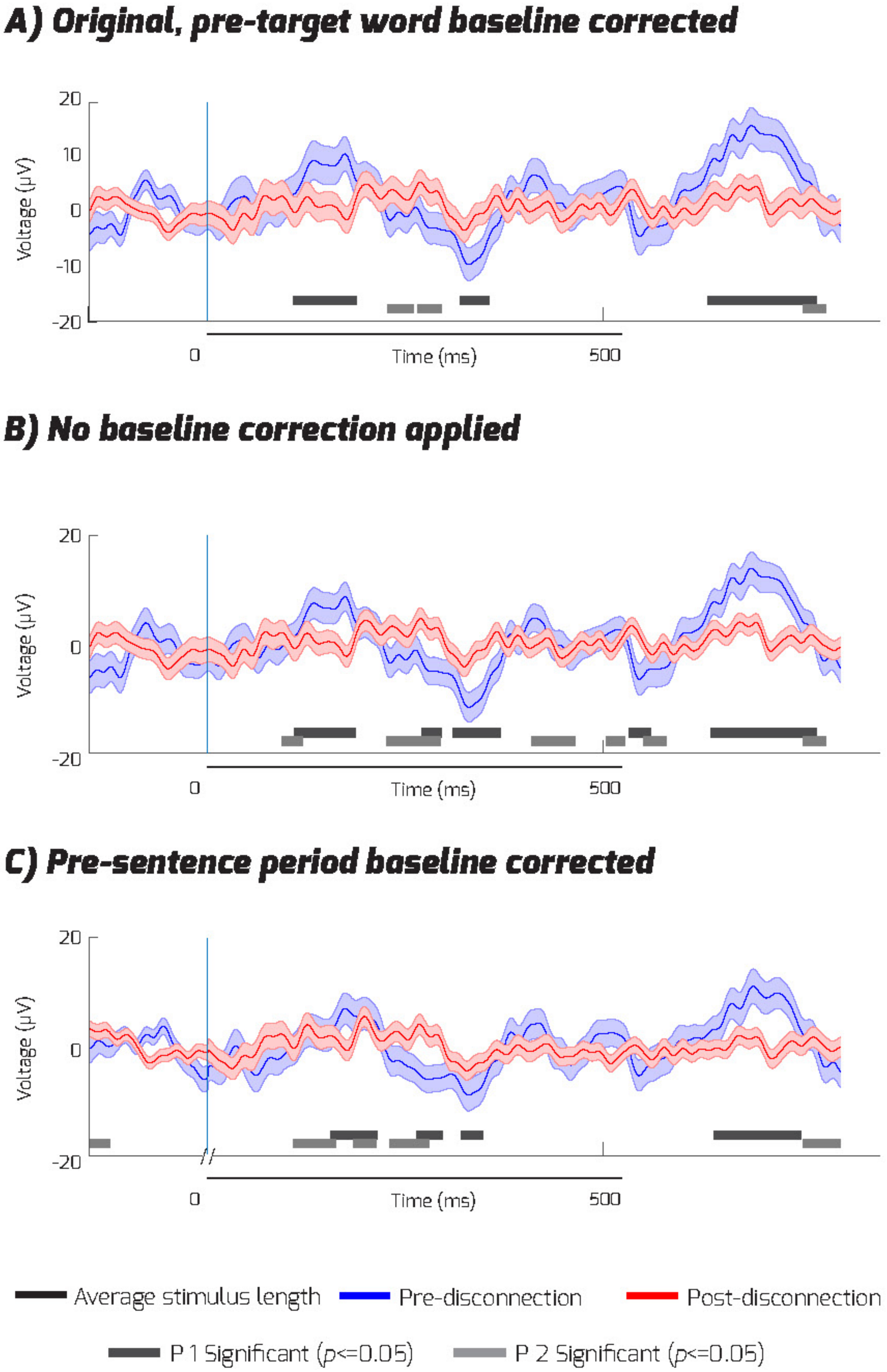

A) Original, pre-target word baseline corrected
20
10
0
-10
-20
Voltage (μV)
0
Time (ms)
500
B) No baseline correction applied
20
0
-20
Voltage (μV)
0
Time (ms)
500
C) Pre-sentence period baseline corrected
20
0
-20
Voltage (μV)
0
Time (ms)
500
Average stimulus length
Pre-disconnection
Post-disconnection
P 1 Significant (p<=0.05)
P 2 Significant (p<=0.05)

**Supplementary Figure 6. Mismatch response evaluated with different baselining methods.** Panel A replicates Fig. 3C to compare to results with other baselining approaches (for this result the canonical baseline correction was used during the pre-target word period (-150 to 0 ms, as per target word onset) in each trial separately for the congruent and incongruent words. B) No baseline correction is applied to the epochs, which shows similar results to the canonical baseline correction. C) Pre-sentence baseline correction: using the baselining method described in A, but on the interval taken from before the onset of the biasing sentence (-150 to 0 ms with respect to sentence onset). The // marks a discontinuity in the x axis, showing that the left of this represents the times taken from before the sentence onset, and the times after this represent the times after target word onset. Figure format: blue traces show the pre-disconnection difference wave between the congruent and incongruent event-related potentials, red traces show the post-disconnection difference wave. The black line denotes the average target word length, the grey lines show the significant period based on the cluster-based permutation testing ($p < 0.05$, 10 000 permutations) for the two participants, separately.

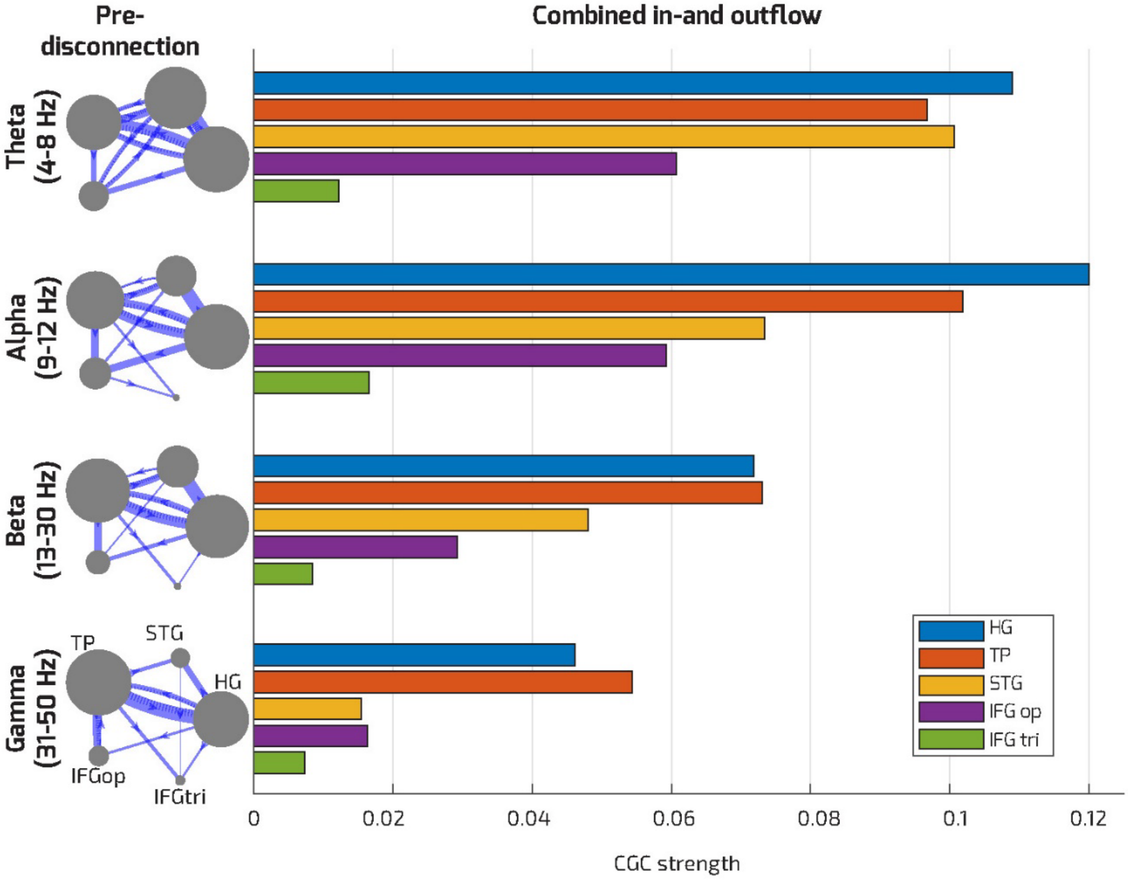


**Supplementary Figure 7. Temporal Pole (TP) pre-disconnection Conditional Granger Causality (CGC) results.** Right: Directions of influence are shown from the regions of interest to recipient regions shown to be active during the target speech sound: HG, STG, TP and IFG pars triangularis and opercularis. Subthreshold (non-significant) regions of time-frequency CGC are masked (set to 0). Left: Combined in- and outward- edge weights from and to each region in each frequency band, showing hub-like activity in TP in higher (beta and gamma) and in HG in lower (theta and alpha) frequency bands. The proportional hubness magnitude for each ROI is shown as gray node size. As noted in the manuscript results, the TP has significantly larger hub strength in the gamma band than the median of the other regions (*p(corrected)* = 0.036). See Methods for the hypothesis and statistical testing approach.

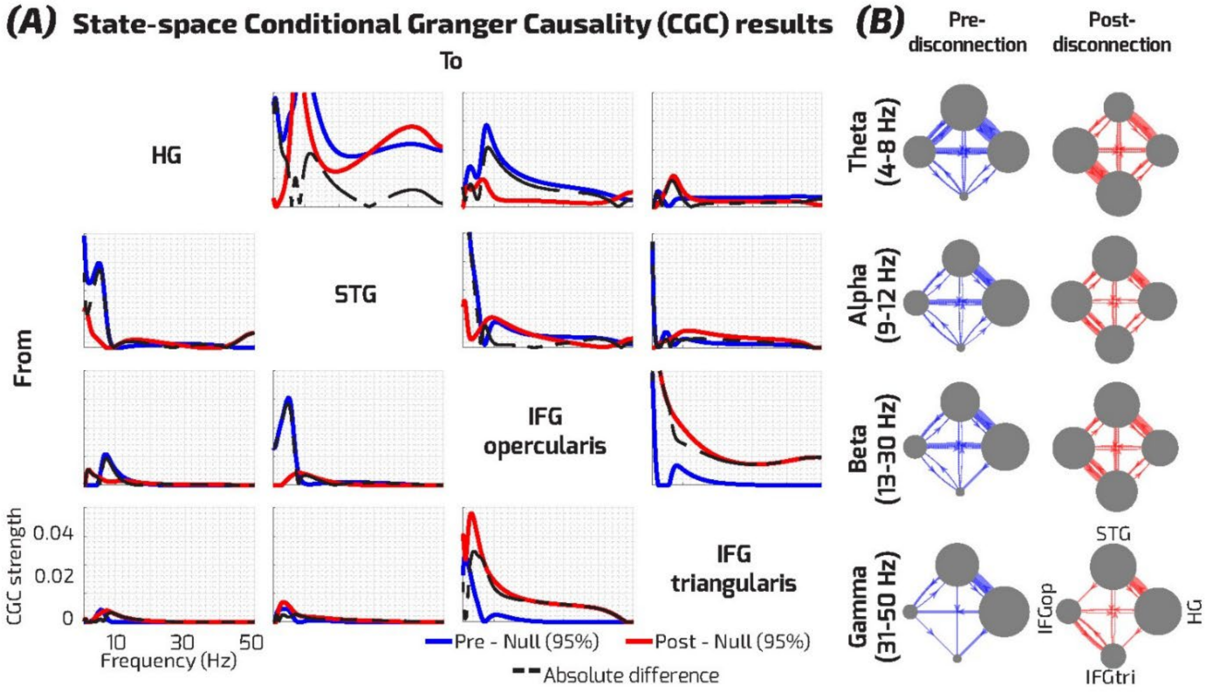


**Supplementary Figure 8. ATL disconnection impact on fronto-temporal network effective connectivity.** (A) Combined frequency-resolved CGC spectral estimates calculated over 0 to 2 seconds relative to the onset of the target word, showing effective connectivity results between the recorded nodes in the fronto-temporal network (HG, STG, IFG p. opercularis and triangularis). Only statistically significant effects are shown for all spectra; the phase-randomized null distribution $95^{th}$ % was subtracted from each of the pre- (blue) and post-disconnection (red) conditions. A permutation test of spectral difference between pre- and post-disconnection is shown in dashed black lines. Directions of influence from regions of interest (rows) to recipient regions (columns) are shown. Note the significantly largely disrupted interconnectivity (blue lines above red) apart from gamma-based interconnectivity in HG to STG and between IFG subregions (red above blue lines). (B) Strong dynamic directional influences are observed between the state-space modeled time-series in HG, STG and the IFG subregions, for the frequency bands (theta, alpha, beta, gamma). The thickness and arrows of the directional edges represents the strength and the direction of the influence (Granger prediction) between ROIs. The proportional hubness magnitude for each ROI is shown as gray node size.

**Supplementary Table 1. Results of logistic mixed effects model for the behavioral task for each participant.** The phoneme identification responses on the semantic prediction task were statistically tested using a logistic mixed effects model with the three within-participant factors: VOT (6 levels: 1 to 6, centered), Semantic bias (2 levels: /b/ and /p/ bias, +/-0.5), and Disconnection (2 levels: before, and 2 months after the surgical procedure, +/-0.5). These were each entered as main effects along with the two-way interactions with disconnection. As these analyses were conducted individually for each subject, the only random factor was Word pair (7 pairs). The combined /b/ and /p/ bias behavioral results for P1 post-disconnection suggest that after the loss of the ATL, P1 adopts a strategy based primarily on the context of the preceding sentence. For P2, the combined /b/ and /p/ behavior after the ATL disconnection procedure suggests a different strategy, less based on the sentential context. However, their behavior is unlikely to be solely a /b/ button response bias because both participants performed very well on the interleaved control trials that require both /p/ and /b/ button responses.

| | **P1** | | | | **P2** | | | |
|---|---|---|---|---|---|---|---|---|
| **Factor** | ***B*** | ***SE*** | ***Z*** | ***p*** | ***B*** | ***SE*** | ***Z*** | ***p*** |
| *VOT* | 3.77 | 0.56 | 6.71 | <0.0001 | 3.32 | 0.75 | 4.45 | <0.0001 |
| *Bias* | 5.95 | 0.87 | 6.83 | <0.0001 | 4.60 | 0.66 | 6.93 | <0.0001 |
| *Disconnection* | 2.06 | 0.60 | 3.44 | 0.0006 | -1.37 | 0.70 | -1.95 | 0.0514 |
| *Disconnection × VOT* | -3.40 | 1.23 | -2.77 | 0.0056 | 0.93 | 1.01 | 0.92 | 0.36 |
| *Disconnection × Bias* | -0.71 | 1.19 | -0.59 | 0.55 | -1.30 | 1.29 | -1.01 | 0.31 |

**Supplementary Table 2. Pre- and post-operative neuropsychological testing scores and calculated Reliable Change Index (RCI) for P1 and P2.** Test abbreviations are as follows: Rey Auditory Verbal Learning Test (RAVLT), Wechsler Adult Intelligence Scale 4th Edition (WAIS-IV), Wechsler Memory Scale 3rd Edition (WMS-III), Trail Making Test (TMT), Lafayette Grooved Pegboard Test (GPT), Boston Naming Test (BNT), Beck Depression Inventory - II (BDI-II), and Beck Anxiety Inventory (BAI). Significant RCI values are indicated in bold italic face type.

| | Test | Subtest | Pre-operative score | Pre-operative Z-score | Post-operative score | RCI |
|---|---|---|---|---|---|---|
| **P1** | RAVLT | Trial 1 | 5 | -0.563 | 4 | -0.533 |
| | | Recall | 8 | -0.200 | 5 | -1.038 |
| | WMS-III | Logical Memory i | 27 | -1.000 | 25 | -0.921 |
| | | Logical Memory II | 12 | -1.000 | 14 | 0.788 |
| | | Faces I | 31 | -1.000 | 38 | ***3.402*** |
| | | Faces II | 29 | -1.000 | 33 | ***1.994*** |
| | | Spatial Span | 12 | -1.000 | 16 | 1.332 |
| | TMT | A | 36 | 0.323 | 25.4 | -0.966 |
| | | B | 56 | -1.194 | 69.8 | 0.440 |
| | BDI | | 18 | N/A | 23 | N/A |
| **P2** | WAIS-IV | Similarities | 9 | -0.333 | 12 | 1.348 |
| | | Digit Span | 9 | -0.333 | 9 | 0.000 |
| | | Arithmetic | 11 | 0.333 | 13 | 1.090 |
| | | Digit Symbol Coding | 8 | -0.667 | 10 | 1.547 |
| | RAVLT | Trial I | 7 | 0.167 | 7 | 0.000 |
| | | Recall | 10 | -0.429 | 8 | -0.692 |
| | WMS-III | Logical memory I | 41 | 0.000 | 39 | -0.921 |
| | | Logical Memory II | 23 | 0.000 | 15 | ***-3.155*** |
| | | Faces I | 25 | -1.667 | 31 | ***2.916*** |
| | | Faces II | 30 | -1.333 | 31 | 0.498 |
| | COWA | | 41 | 0.418 | 56 | 1.907 |
| | BNT | | 55 | -0.580 | 57 | 0.722 |
| | GPT | Dominant | 78 | 1.792 | 74 | -0.385 |
| | | Nondominant | 89 | 1.791 | 77 | -0.787 |
| | TMT | A | 21 | -0.390 | 30 | 0.820 |
| | | B | 43 | -0.621 | 61 | 0.472 |
| | BDI | | 30 | N/A | 8 | N/A |
| | BAI | | 30 | N/A | 17 | N/A |

**Supplementary Table 3. VOT peak times (ms) on the averaged and individual ERPs on the Heschl's gyrus electrodes pre- and post-disconnection.**

| VOT level pre-disconnection (ms) | Average Peak 1 | Average Peak 2 | Participant 1 Peak 1 | Participant 1 Peak 2 | Participant 2 Peak 1 | Participant 2 Peak 2 |
|---|---|---|---|---|---|---|
| 0 | 266 | 322 | 272 | 324 | 243 | 294 |
| 8 | 269 | 312 | 272 | 335 | 252 | 309 |
| 16 | 268 | 329 | 272 | 351 | 261 | 316 |
| 24 | 279 | 332 | 288 | 340 | 265 | 325 |
| 32 | 281 | 339 | 289 | 362 | 275 | 334 |
| 40 | 292 | 340 | 295 | 343 | 287 | 337 |

| VOT level post-disconnection (ms) | Average Peak 1 | Average Peak 2 | Participant 1 Peak 1 | Participant 1 Peak 2 | Participant 2 Peak 1 | Participant 2 Peak 2 |
|---|---|---|---|---|---|---|
| 0 | 257 | 315 | 261 | 319 | 253 | 312 |
| 8 | 269 | 320 | 270 | 354 | 252 | 313 |
| 16 | 270 | 330 | 275 | 342 | 259 | 322 |
| 24 | 283 | 332 | 285 | 356 | 275 | 329 |
| 32 | 293 | 341 | 292 | 343 | 292 | 335 |
| 40 | 294 | 347 | 297 | 363 | 291 | 343 |

**Supplementary Table 4. Vital signs for the two participants during the intracranial recording periods and anesthesia drug stoppage before the awake recordings**

| | **Participant 1** | | **Participant 2** | |
|---|---|---|---|---|
| | **Pre-disconnection** | **Post-disconnection** | **Pre-disconnection** | **Post-disconnection** |
| *Time of recording* | 11:34 – 12:03 | 14:48 – 15:15 | 12:38 – 13:07 | 15:03 – 15:34 |
| *Dexmedetomidine stopped* | 10:59 – 13:24<br>(35 minutes before testing period) | 14:25 – 15:24<br>(23 minutes before testing period) | 11:09 – 13:27<br>(37 minutes before testing period) | 14:40 – 15:34<br>(23 minutes before testing period) |
| *Heart rate (average beats per minute; +/- SD) during recording period* | 59.19 (1.28) | 66.93 (3.25) | 58 (3.91) | 55.94 (1.77) |
| *Respiration rate (average/minute; +/- SD) during the recording period* | 16.25 (0.51) | 18.59 (1.02) | 15.71 (2.6) | 13.45 (4.12) |

## Supplementary Table 5. Detailed description of the experimental stimuli.

| Experimental trials | | | | |
|---|---|---|---|---|
| *Target word pair* | *Bias* | *Sentence* | *Times played during experiment* | *Total number of trials* |
| **Back/pack** | B | She stole my doll, so I asked her to give it.... | 12 | 72 |
| | B | Don't worry, I got your... | 12 | |
| | B | I can't reach to scratch my... | 12 | |
| | P | A school is to fish what to wolves is a... | 12 | |
| | P | For school, I need a back... | 12 | |
| | P | I ran out of cigarettes, so I'll go buy a... | 12 | |
| **Beach/peach** | B | He enjoyed the ocean air, so he often went to the... | 12 | 72 |
| | B | She lied on the sandy... | 12 | |
| | B | While in LA we went to Venice... | 12 | |
| | P | Super Mario found Princess... | 12 | |
| | P | The state fruit of Georgia is the... | 12 | |
| | P | Isn't she just a Georgia... | 12 | |
| **Bad/pad** | B | There are some good news and some... | 12 | 72 |
| | B | Well, that's too... | 12 | |
| | B | Sometimes he's good, sometimes he's... | 12 | |
| | P | You move the computer mouse on a mouse... | 12 | |
| | P | In the lake we saw a lily... | 12 | |
| | P | The i-phone is smaller than an i-... | 12 | |
| **Bark/park** | B | The dog started to... | 12 | 72 |
| | B | The outer part of a tree is called... | 12 | |
| | B | Quiet dogs sometimes also... | 12 | |
| | P | There are several roller coasters in that amusement... | 12 | |
| | P | Driving in Iowa City is miserable because there is never anywhere to... | 12 | |
| | P | In New York there is the Central... | 12 | |
| **Bath/path** | B | If you are dirty, you should get in the tub and take a... | 12 | 72 |
| | B | She took a nice warm... | 12 | |
| | B | The little girl took a bubble... | 12 | |
| | P | She was led down the garden... | 12 | |
| | P | Unfortunately, our shop is a bit off the beaten... | 12 | |
| | P | He went down the wrong... | 12 | |
| **Bill/pill** | B | The governor vetoed the... | 12 | 72 |
| | B | I paid my water... | 12 | |
| | B | In order to register, you must pay your university... | 12 | |
| | P | Relax and take a chill... | 12 | |
| | P | I order to sleep I take a sleeping... | 12 | |
| | P | If you are in pain, take a pain... | 12 | |
| **Bowl/pole** | B | I poured my cereal into the... | 12 | 72 |
| | B | Beyonce sang at the super... | 12 | |
| | B | We're going to Florida to watch the Orange... | 12 | |
| | P | Santa lives at the North... | 12 | |
| | P | Greenland is right under the North... | 12 | |
| | P | There are no penguins in the South... | 12 | |
| **Total number of experimental trials** | | | | 504 |

| Filler trials | | |
|---|---|---|
| *Filler word* | *Sentence* | *Total number of trials* |
| **Bar** | A pub is just another name for a... | 126 (randomly drawn) |
| | I'll just get something from the salad… | |
| | If you want a drink, you should go to a... | |
| **Boat** | Whatever floats your... | |
| | We went fishing on my dad's... | |
| | I am bored of sailing; I want a motor... | |
| **Bone** | Every Thanksgiving, my sister and I fight over the wish... | |
| | I've sprained my ankle, but I've never broken a... | |
| | The dog ran out to bury her... | |
| **Book** | He's so predictable, like an open... | |
| | On kindle you can read an e-... | |
| | Let me read my... | |
| **Boots** | My feet stayed dry because I wore my rain... | |
| | This dress goes with these cowboy... | |
| | It's snowing so put on a pair of snow... | |
| **Peace** | Miss Universe wished for world... | |
| | Speak up or forever hold your... | |
| | War is the opposite of... | |
| **Pope** | The head of the Catholics church is the... | |
| | In the Vatican they elected the new... | |
| | He wore a tall white hat and a big cross just like the... | |
| **Pen** | I need a pencil or a... | |
| | There is no more ink in this... | |
| | The pitcher just went into the bull... | |
| **Pain** | I need an Advil because I'm in... | |
| | I'm completely numb, I feel no... | |
| **Plate** | You just need to step up to the... | |
| **Paint** | The walls need another coat of... | |
| | They're close as two coats of... | |
| **Pros** | She made a list with cons and... | |

| Catch trials | | | *Total number of trials* |
|---|---|---|---|
| Any of the experimental trials played except for the target word | Target word appears on screen with counterpart | E.g., Participant listens to sentence: “I can’t reach to scratch my…”<br>Visually presented:<br>back ● pack | 63 (randomly drawn) |

**Supplementary Table 6. Detailed results for the hubness strength of TP electrodes versus the median of the other 4 regions of interest (HG, STG, IFG opercularis, IFG triangularis).** To statistically evaluate the reliability of the connectivity results we used a phase-randomization surrogate data technique to construct an empirical null distribution representing chance influence between ROIs. This method consists of randomly shuffling the Fourier phases of each of the ECoG recordings which generates uncorrelated data with preserved autocorrelation properties. The matrix of spectral CGC between four ROIs in HG, STG and IFG opercularis and triangularis was statistically evaluated as follows: 2,000 surrogates were generated and thresholded at $\alpha$ = 0.05 of the null distribution, values below which were subtracted from the observed spectral CGC and trimmed at zero. Permutation tests were performed for each frequency band by constructing a null distribution of signed pre- and post-disconnection spectrally segmented differences following recalculation of spectral CGC after random assignment of time series to the two categories. To compare whether directional influences between ROIs change between pre- and post-disconnection, random permutation tests were performed on the absolute difference between spectral CGC across frequencies. Each post-stimulus trial time series was randomly assigned to either the pre- or post-disconnection category and spectral CGC recalculated 2,000 times. An empirical null distribution was calculated by summing the absolute CGC differences between pre- and post-disconnection across frequencies. The number of permuted surrogates that exceeded the measured difference was used to calculate *p*-values. Multiple comparison correction was applied: false discovery rate (FDR) was controlled at the 0.05 level across ROI pairs and frequency bands.

| **Frequency band** | **Uncorrected p value** | **Corrected p value** |
|---|---|---|
| **Theta** | 0.302 | 0.302 |
| **Alpha** | 0.156 | 0.207 |
| **Beta** | 0.081 | 0.163 |
| **Gamma** | 0.009 ** | 0.036* |

* Significant with FDR correction for multiple comparisons (Methods)

**References:**

1. Yeh, F.-C., Verstynen, T. D., Wang, Y., Fernández-Miranda, J. C. & Tseng, W.-Y. I. Deterministic Diffusion Fiber Tracking Improved by Quantitative Anisotropy. *PLOS ONE* **8**, e80713 (2013).
