# Supplementary material for: Immediate neural impact and incomplete compensation after semantic hub disconnection
Source: Nat Commun. 2023 Oct 7;14:6264. doi: 10.1038/s41467-023-42088-7 (PMC10560235; doi:10.1038/s41467-023-42088-7)
Supplement: Supplementary file 3 — Reporting Summary [file 41467_2023_42088_MOESM3_ESM.pdf]

nature portfolio

Corresponding author(s): Zsuzsanna Kocsis, Christopher I. Petkov

Last updated by author(s): Sep 1, 2023

Reporting Summary

Nature Portfolio wishes to improve the reproducibility of the work that we publish. This form provides structure for consistency and transparency in reporting. For further information on Nature Portfolio policies, see our Editorial Policies and the Editorial Policy Checklist.

Statistics

For all statistical analyses, confirm that the following items are present in the figure legend, table legend, main text, or Methods section.

n/a	Confirmed	
☐	☒	The exact sample size (*n*) for each experimental group/condition, given as a discrete number and unit of measurement
☐	☒	A statement on whether measurements were taken from distinct samples or whether the same sample was measured repeatedly
☐	☒	The statistical test(s) used AND whether they are one- or two-sided<br>*Only common tests should be described solely by name; describe more complex techniques in the Methods section.*
☐	☒	A description of all covariates tested
☐	☒	A description of any assumptions or corrections, such as tests of normality and adjustment for multiple comparisons
☐	☒	A full description of the statistical parameters including central tendency (e.g. means) or other basic estimates (e.g. regression coefficient) AND variation (e.g. standard deviation) or associated estimates of uncertainty (e.g. confidence intervals)
☐	☒	For null hypothesis testing, the test statistic (e.g. *F*, *t*, *r*) with confidence intervals, effect sizes, degrees of freedom and *P* value noted<br>*Give P values as exact values whenever suitable.*
☒	☐	For Bayesian analysis, information on the choice of priors and Markov chain Monte Carlo settings
☒	☐	For hierarchical and complex designs, identification of the appropriate level for tests and full reporting of outcomes
☐	☒	Estimates of effect sizes (e.g. Cohen's *d*, Pearson's *r*), indicating how they were calculated

Our web collection on statistics for biologists contains articles on many of the points above.

Software and code

Policy information about availability of computer code

Data collection	MATLab 2018b
Data analysis	MATLab 2018b, EEGLab 14_1_2b, FSL v5.0, FreeSurfer v6.0

For manuscripts utilizing custom algorithms or software that are central to the research but not yet described in published literature, software must be made available to editors and reviewers. We strongly encourage code deposition in a community repository (e.g. GitHub). See the Nature Portfolio guidelines for submitting code & software for further information.

Data

Policy information about availability of data

All manuscripts must include a data availability statement. This statement should provide the following information, where applicable:

- Accession codes, unique identifiers, or web links for publicly available datasets
- A description of any restrictions on data availability
- For clinical datasets or third party data, please ensure that the statement adheres to our policy

The datasets generated in this study have been deposited in the Zenodo database under accession code 10.5281/zenodo.8110724.

Research involving human participants, their data, or biological material

Policy information about studies with human participants or human data. See also policy information about sex, gender (identity/presentation), and sexual orientation and race, ethnicity and racism.

Reporting on sex and gender	No reporting on sex and gender is possible.
Reporting on race, ethnicity, or other socially relevant groupings	No reporting on race, ethnicity, or other socially relevant groupings is possible.
Population characteristics	2 neurosurgical patient procedures and pre -and post-surgical recordings (P1: 63-year-old woman, P2: 32-year-old man). The patients had been clinically diagnosed with medically refractory drug-resistant epilepsy and were undergoing a one day acute surgical procedure to remove seizure foci requiring anterior temporal lobe disconnection, during clinical monitoring and awake intracranial recordings with speech and motor clinical mapping in the operating room, as reported in the paper.
Recruitment	Neurosurgery patients scheduled to undergo seizure focus resection who were above 18 years and below 75 years of age and who demonstrated capacity to consent were eligible to participate in this research. The patients were approached by the principal investigator (Howard) and/or other research team members (Kocsis, Rhone) during an outpatient neurosurgery clinic visit prior to their surgery. The research team read the consent document, explained the study procedures, and answered questions. The two participants provided written informed consent to participate in this study, following the latest ethical guidance procedures for intracranial recording research with neurosurgery patients.
Ethics oversight	University of Iowa Institutional Review Board ethical approval, IRB #200112047

Note that full information on the approval of the study protocol must also be provided in the manuscript.

Field-specific reporting

Please select the one below that is the best fit for your research. If you are not sure, read the appropriate sections before making your selection.

☒ Life sciences ☐ Behavioural & social sciences ☐ Ecological, evolutionary & environmental sciences

For a reference copy of the document with all sections, see nature.com/documents/nr-reporting-summary-flat.pdf

Life sciences study design

All studies must disclose on these points even when the disclosure is negative.

Sample size	This is a within subjects study. Sample size was limited to these two patient procedures.
Data exclusions	No data were excluded. The recording contacts included for analysis following preprocessing of all data were those that showed significant speech responses anywhere in the fronto-temporal regions recorded in both patients.
Replication	We ensured that all main interpretations are based on significant and consistent effects in both participants. Within subject replication was also assessed across recording contacts within and across both participants, and generally between the pre- and post-disconnection samples for properties not expected to differ.
Randomization	The stimulus conditions were randomly sampled and presented. Permutations tests randomized conditions for significance testing.
Blinding	It was not possible for the investigators to be blinded to the patient contributing the samples during the experiment. However, data analyses pipelines were conducted in the same way for both pre- and post-disconnection datasets comparisons to reduce experimental bias.

Reporting for specific materials, systems and methods

We require information from authors about some types of materials, experimental systems and methods used in many studies. Here, indicate whether each material, system or method listed is relevant to your study. If you are not sure if a list item applies to your research, read the appropriate section before selecting a response.

Materials & experimental systems

n/a	Involved in the study
☒	☐ Antibodies
☒	☐ Eukaryotic cell lines
☒	☐ Palaeontology and archaeology
☒	☐ Animals and other organisms
☒	☐ Clinical data
☒	☐ Dual use research of concern
☒	☐ Plants

Methods

n/a	Involved in the study
☒	☐ ChIP-seq
☒	☐ Flow cytometry
☐	☒ MRI-based neuroimaging

Magnetic resonance imaging

Experimental design

Design type: N/A

Design specifications: N/A

Behavioral performance measures: N/A

Acquisition

Imaging type(s): Diffusion and structural MRI sequences

Field strength: 3T

Sequence & imaging parameters: T1-weighted structural MRI scans (fast spoiled gradient-echo sequence, 1x1x0.8 mm resolution, TR=8.508, TE=3.288, flip angle=12°, 32 slices)

Area of acquisition: Whole brain scans

Diffusion MRI: ☒ Used ☐ Not used

Parameters: 64 direction diffusion-weighted MRI (1x1x2 mm resolution, TR=10332, TE=88.9, flip angle=90°)

Preprocessing

Preprocessing software: FreeSurfer version 6.0 using the recon-all, default parcellation scheme from FreeSurfer (the Desikan-Killiany atlas69,70), with 82 anatomical cortical and subcortical ROIs.

Normalization: Data were only whole brain intensity corrected but not otherwise normalized. Imaging scans were brain extracted using (FSL BET) then the post-operative and the pre-operative images were stepwise registered (6 degrees of freedom) to a common space in each subject using FSL FLIRT.

Normalization template: No template was used, the images were co-registered to each individual's common space.

Noise and artifact removal: Susceptibility distortions on diffusion-weighted MRI data were corrected using the SynB0 DisCo tool in conjunction with the FSL TOPUP tool. The FSL tool 'EDDY' was used to correct for motion and eddy current distortions with the bvecs rotated appropriately.

Volume censoring: N/A

Statistical modeling & inference

Model type and settings: N/A

Effect(s) tested: N/A

Specify type of analysis: ☐ Whole brain ☒ ROI-based ☐ Both

Anatomical location(s): N/A

Statistic type for inference (See Eklund et al. 2016): N/A

Correction: N/A

Models & analysis

n/a	Involved in the study
☐	☒ Functional and/or effective connectivity
☐	☒ Graph analysis
☒	☐ Multivariate modeling or predictive analysis

Functional and/or effective connectivity: N/A

Graph analysis: N/A
